# Evaluation of a Digital Self-management Platform for Patients With Chronic Illness in Primary Care: Qualitative Study of Stakeholders’ Perspectives

**DOI:** 10.2196/38424

**Published:** 2022-08-03

**Authors:** Steven van de Vijver, Deirdre Hummel, Annericht Hester van Dijk, Jan Cox, Oscar van Dijk, Nicoline Van den Broek, Esther Metting

**Affiliations:** 1 Amsterdam Health & Technology Institute Amsterdam University Medical Center Amsterdam Netherlands; 2 Family Medicine Department OLVG Amsterdam Netherlands; 3 Faculty of Economics and Business University of Groningen Groningen Netherlands; 4 Medicine Men Utrecht Netherlands; 5 Department of General Practice and Elderly Medicine University Medical Center Groningen University of Groningen Groningen Netherlands; 6 Data Science Center in Health University Medical Center Groningen University of Groningen Groningen Netherlands

**Keywords:** primary care, chronic disease, telemonitoring, digital health, self-management, patient-centered care, chronic care, chronic care management, illness, healthcare, healthcare professional, user, patient, platform, tool, communication, empowerment, online

## Abstract

**Background:**

Population aging and multimorbidity has led to increasing chronic care needs associated with new challenges in managing growing costs, rising health care professional workloads, and the adoption of rigorous guidelines. These issues could all benefit from greater digitalization and a more patient-centered approach to chronic care, a situation brought to the fore by the COVID-19 pandemic. Little is known about real-life use in primary care.

**Objective:**

This study aimed to explore the views, thoughts, usability, and experiences concerning a recently introduced digital self-care platform for chronic conditions in 3 Dutch primary care practices.

**Methods:**

We conducted an explorative study combining questionnaires and interviews among patients and general practitioners from 3 general practices that used the digital platform. Questionnaires were sent to patients in each practice to seek the views and experiences of both patient nonusers (n=20) and patient users (n=58) of the platform, together with standardized questionnaires about illness perception and quality of life. In addition, patients (n=15) and general practitioners (n=4) who used the platform took part in semistructured interviews. We transcribed interviews verbatim and performed qualitative content analysis using a deductive approach. The results of the questionnaires were analyzed with descriptive analysis.

**Results:**

Among patients who had not actively used the platform but had received an explanation, only 35% (7/20) would recommend its use due to concerns over communication and handling. However, this percentage increased to 76.3% (45/59) among the people who actively used the platform. Interviews with patients and general practitioners who used the platform uncovered several key benefits, including reduced time requirements, reduced workload, improved care quality, and improved accessibility due to the greater patient-centeredness and use of different communication tools. In addition, the self-management tool led to greater patient autonomy and empowerment. Although users considered the platform feasible, usable, and easy to use, some technical issues remained and some patients expressed concerns about the reduction in human contact and feedback.

**Conclusions:**

The overall experience and usability of the platform was good. Support for the online self-management platform for chronic care increased when patients actively used the tool and could experience or identify important advantages. However, patients still noted several areas for improvement that need to be tackled in future iterations. To ensure benefit in the wider population, we must also evaluate this platform in cohorts with lower digital and health literacy.

## Introduction

Western health care systems are facing challenges due to population aging and the rising number of chronic diseases [1,2]. Crucially, these chronic diseases are costly for individuals and the health care system, representing an important limiter of life quality [3,4], and are responsible for increased workloads experienced by general practitioners (GPs) [5]. Traditional clinical pathways offer standardized care that can be seen as rigid when physicians make decisions for their patients based on strict guidelines, and this can result in poor adherence and ownership by patients [5-7]. However, this approach contradicts modern views that patients should be responsible for their health and lifestyle [7]. Health care systems require a shift toward patient-centered care, with prevention and health lead by patients themselves [8].

Patient-centered care considers the individual preferences, needs, and values necessary to guide all clinical decisions [9] to improve quality of life for patients [10]. To achieve patient-centered care, patients must also engage in self-management and shared decision-making. Self-management requires that patients with chronic disease manage their own symptoms, treatment, lifestyle changes, and any consequences [6]. This can empower patients by increasing their autonomy [11], and it benefits by offering direct feedback via the required self-monitoring of vital signs [12]. Shared decision-making is a process through which health care providers make important decisions with patients regarding disease management or a lifestyle change [13].

Digital health can facilitate the change to patient-centered care by offering web-based information programs, remote monitoring, teleconsultations, and home care supported by mobile devices [14]. Health outcomes when comparing these services with in-person consults to date have either been similar [9,15] or have shown improvement [16]. Digital health can also improve health care effectiveness, efficiency, and accessibility in the context of population aging and greater disease chronicity [12], helping to manage increased GP workloads [17] while still offering patient-centered care [18]. The COVID-19 pandemic led to a marked increase in digital health implementations [19], consistent with models that predict health behavior and acceptance of technology, such as the health belief model [20] and the unified theory of acceptance and use of technology (UTUAT) model [21], respectively.

We implemented an online self-management platform for patients living with chronic illness in 3 Dutch primary care facilities. In this study, we aim to describe this innovative approach and evaluate (1) views on care digitalization and intention to use the new platform among patients who did not use the platform questionnaires, (2) experiences with the platform and thoughts about illness and quality of life among patients who used the platform questionnaires, and (3) experiences of GPs and patients who used the platform.

## Methods

### Online Platform

Three Dutch primary care practices (Westerdokters, Veendokters, and Wouwse Markt), all members of the Flexdokters Cooperative, introduced a digitally supported self-care platform in April 2020. Designed by GPs from Flexdokters as a tailor-made service for patients with chronic illness, it used the online Viduet platform (Medicine Men), an independent tool to monitor chronic diseases that is partly integrated with the electronic health information system of the GP. It does not contain all medical information of the patient, only data that are relevant for chronic diseases like, for example, blood pressure and glucose levels. The costs are mainly covered by health insurance and offered for free to participating patients. GPs can recommend the platform to eligible patients and offer an initial explanation via video call or in-person contact. Shared decision-making is then used to select appropriate measuring devices, symptom questionnaires, contact options, and measurement frequency. If they agree, patients receive the necessary devices and user manuals at their home address and a link by email to create a platform account. Patients can then use validated questionnaires to monitor asthma, chronic obstructive pulmonary disease, cardiovascular risk, diabetes, depression, or other chronic diseases. Available devices include blood pressure monitors, glucose meters, oxygen saturation meters, smartwatches, fitness trackers, thermometers, and weighing scales, which are sent if needed.

Data are input manually or automatically (via Bluetooth). The platform offers access to a technical help desk for health care professionals and patients that refers any medical questions from patients to the health care provider. The GP retains control over a given patient’s participation, but that patient has full autonomy over their care. Patients must grant permission for GPs to access their data via a viewer in the electronic patient record, which can also give alerts if measurements are above or below certain predetermined thresholds or if data collection stops.

### Study Design

This explorative study comprised 3 elements (see Table 1). All patients with known chronic care needs, irrespective of platform use, received an email invitation from their GP asking if they would participate in the study, and if agreeable, to return an included questionnaire and informed consent form. Thereafter, nonusers received a combined questionnaire covering their disease status and thoughts about the platform, while users received a Dutch revised version of the Illness Perception Questionnaire–Short (IPQ-K) [22,23], the 12-Item Short Form Survey (SF-12) [24] for quality of life, and the System Usability Scale (SUS) [25].

We used convenience sampling to include participants based on time and availability to respond during a 2-month period. Participants also needed to be able to speak Dutch or English and could have no disability that might limit their ability to answer questions. The research team included 2 GPs, one epidemiologist/psychologist, 2 information technology (IT) experts, and 3 research assistants. Two master’s in business administration in health students (research assistants) at the University of Groningen received training in qualitative research and performed the interviews.

**Table 1:** Study design.

Element	Sample, n	Participants and methods	Aim
1	20	Patients in the practice who do not use the platform questionnaire	Evaluation of views on digitalization of care and intention to use the new platform
2	61 partly 58 fully	Patients who use the platform questionnaires (IPQ-K^a^ for illness perception, SF-12^b^ for quality of life, and SUS^c^ for usability)	Evaluation of their experiences with the platform and their thoughts toward illness and quality of life
3	19	Semistructured interviews with patients (n=15) and general practitioners (n=4) who used the platform	Evaluation of their experiences with the platform

^a^IPQ-K: Illness Perception Questionnaire–Short.

^b^SF-12: Short Form Survey–12 Item.

^c^SUS: System Usability Scale.

### Ethics Approval

The University of Groningen approved this research (20200060047). Each participant completed an informed consent form that included their rights and what to expect prior to inclusion. Participation was voluntary and data were anonymized during collection. Identifiable information were kept to a minimum, stored on a safe drive at the university, and only used for this study.

### Data Collection

#### Questionnaires

Questionnaires were sent by GPs via the Qualtrics system for online questionnaires from December 2020 to January 2021, and participants could complete and upload them online.

To understand how relative outsiders viewed the platform, patients who had not used it were asked about their digital activity in daily life and their opinion of the platform after receiving information about its aims and content (Multimedia Appendix 1). We used multiple choice and 7-point Likert scales and recorded their age, sex, and chronic disease to enable comparison with users.

To clarify the practical aspects of the platform, users were asked to provide an evaluation by completing the SUS. In addition, the IPQ-K and SF-12 standardized questionnaires were completed, and we obtained data on age, sex, chronic disease, and health-related quality of life.

#### Interviews

Patients and GPs who used the platform were interviewed by phone until saturation (15 patients) or until no additional participants were available (4 GPs). The interviews included general questions about IT and digital health, personal experiences with the platform, effort expectancy, user intentions, barriers, and facilitators. Factors were chosen based on the health belief model and the UTUAT model. Quotes from interviews are used to illustrate the study findings.

#### Data Analysis

Questionnaire data were analyzed descriptively using proportions and numbers. All interviews were recorded and transcribed verbatim. Thematic and axial coding were used to analyze the qualitative data, starting with the main interview themes, before performing deductive coding to look for patterns in the transcripts. ATLAS.ti (ATLAS.ti Scientific Software Development GmbH) was used for coding.

## Results

### Element 1: Questionnaire Responses of Platform Nonusers

In total, 20 patients (55% female, average age 69 [SD 9] years) completed the questionnaire. The main chronic diseases were hypertension/cardiovascular disease, chronic respiratory disease, and a few individual cases of thyroid disease and prostate hypertrophy. Age, sex, and chronic disease characteristics were comparable with users.

All respondents had access to the internet at home, with 85% (17/20) using laptops or smartphones, 60% (12/20) using tablets, and 50% (10/20) using desktop computers at least weekly. When asked about their digital skills, one considered computer use and two considered smartphone use a bit difficult, while the remaining 85% (17/20) considered themselves digitally skilled. Two respondents considered technology a bad development for health care because they wanted “to be treated as individual and not as robot” or “to have a GP in front of me to spar together.” Those who thought it was a good development liked the speed and efficiency of the process for patients and GPs, stating “it can be practical and reduce time to have digital consultations.”

According to 35% (7/20) of respondents, they expected that communication with the health care professional would change when using the platform. One did not expect that the information provided would be treated confidentially, while 40% (8/20) wanted to share their data in the platform with other health care professionals. The following free-text comment was made regarding data exchange:

I have reservations. Not so much about the app itself, but...about the handling, data processing, and follow-up. Treatment and cure is more than collecting data.

Patient

Overall, however, slightly more than a third (35%) would recommend the platform after they received an explanation of its functions.

### Element 2: Questionnaire Responses of Platform Users

Among the patients who used the platform, 61 partially completed and 58 fully completed the 3 questionnaires (33/61, 54.1% female, average age 62 [SD 8] years).

#### IPQ-K Instrument

**Figure 1:**
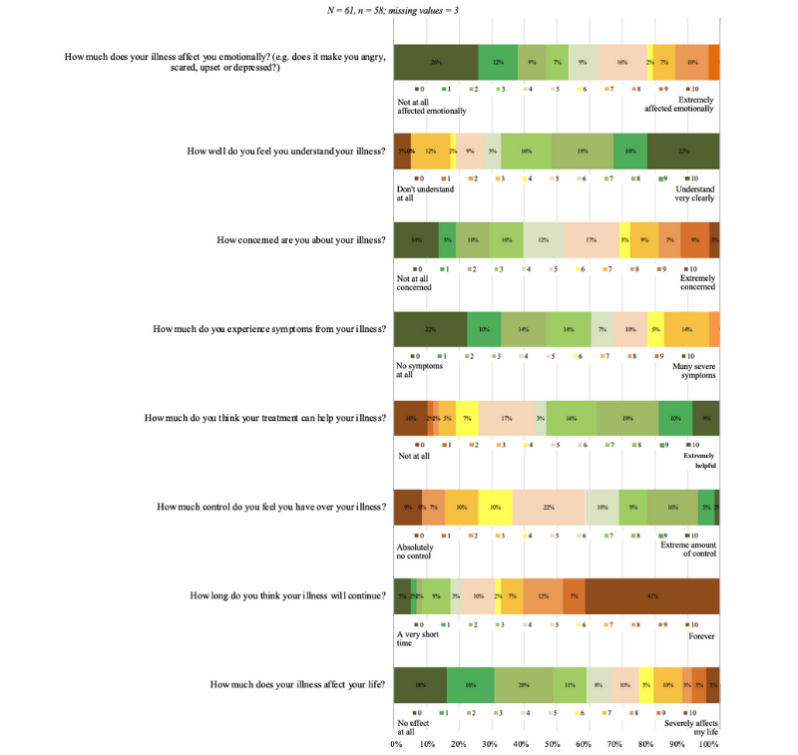
Results of Illness Perception Questionnaire–Short.

Users more often reported that they are largely unaffected by symptoms, will have them for life, have some control over them, know that treatment helps, and that they cause only moderate worry (Figure 1). They typically reported having a good understanding of their complaints and that these did not severely influence their mood. In general, the complaints had a relatively minor impact on patients. The 3 most frequently mentioned causes of their complaints were stress, heredity/genes, and lifestyle habits (eg, overeating, alcohol, and lack of exercise), while events (eg, COVID-19 or an accident) or mental/physical conditions were mentioned less often.

#### SF-12 Instrument

**Figure 2:**
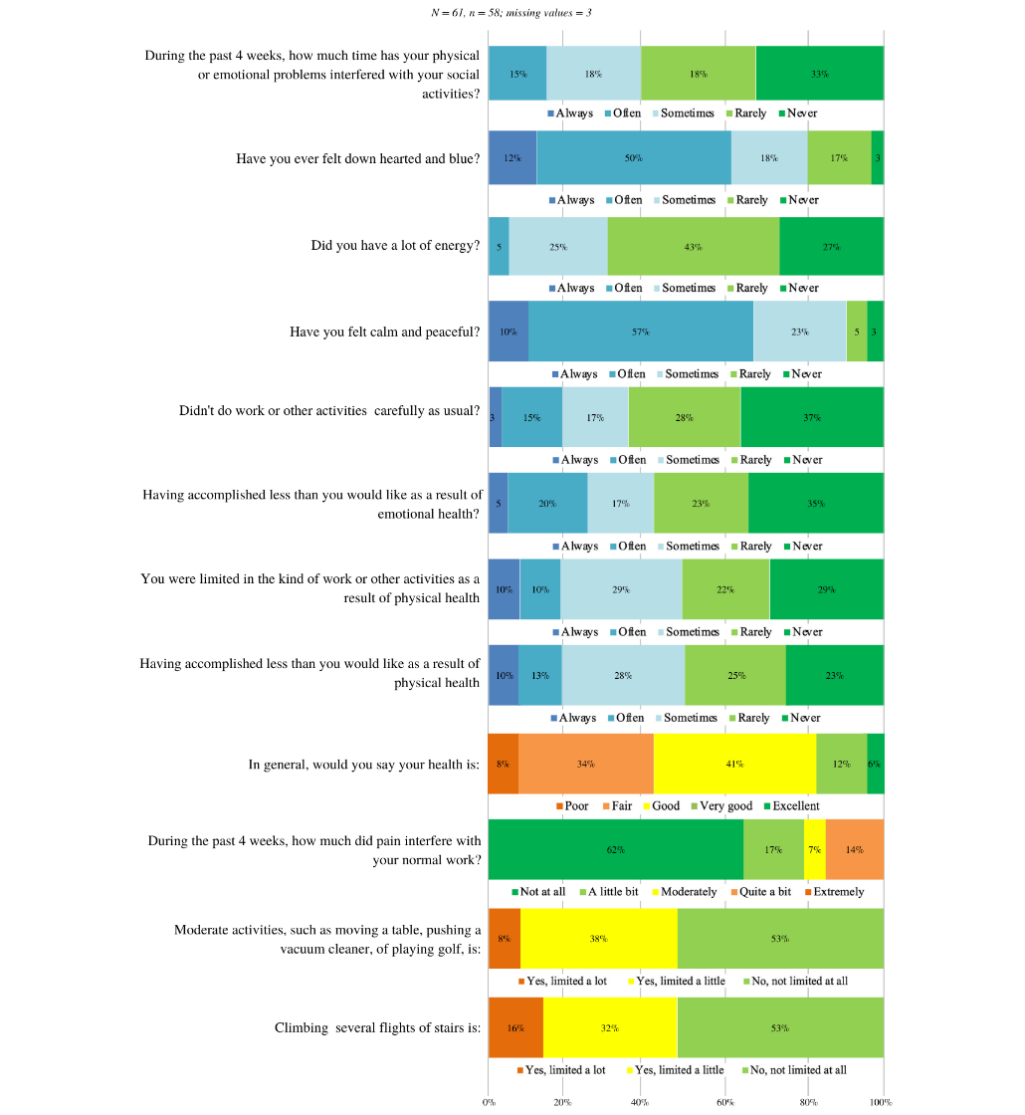
Short Form Survey–12 Item: summary of responses for health-related quality of life among platform users.

Figure 2 summarizes the questions and responses concerning health-related quality of life among platform users. Patients who used the platform rated their health from poor to excellent, with most considering it fair or good. Over half (30/59, 50.8%) indicated that they were not limited at all when engaging in moderate levels of exertion, with most users considering themselves somewhat or not limited by their symptoms. Regarding how health affected work and other daily activities, the distribution varied from sometimes to never being affected. Moreover, users were typically never or rarely bothered by their mental health, while most were not hindered at all and none were hindered very much by pain. In the 4 weeks preceding the questionnaire, users typically reported that they felt calm and content, had a lot of energy, did not feel gloomy or dejected, and were rarely or never hindered by their physical or emotional health during social activities.

#### SUS Instrument

**Figure 3:**
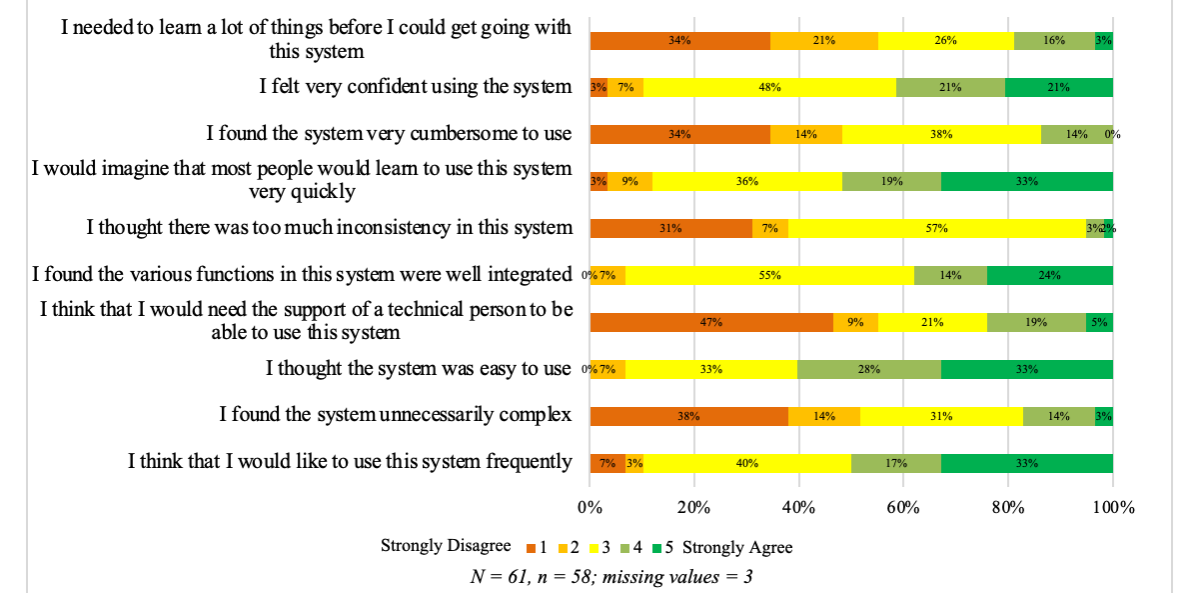
System Usability Scale: summary of responses among platform users.

Users indicated the extent to which they agreed or disagreed with each of 10 statements (Figure 3). Only 10.1% (6/59) of users stated that they will not use the platform regularly, with 6.8% (4/59) answering that it was not easy to use. Almost a quarter (14/59, 23.7%) of users mentioned that they would like to receive external support while using the platform. Users were neutral on the statements regarding how well functions were integrated (33/60, 55.0%), the presence of inconsistencies (34/60, 56.7%), whether most people will learn to use the platform quickly (21/59, 35.6%), whether the platform is too cumbersome (23/60, 38.3%), and whether they felt confident using the platform (29/60, 48.3%). However, users typically disagreed completely with the statements that the platform was unnecessarily complicated (23/60, 38.3%), that they needed technical support (28/59, 47.4%), or that they had to learn a lot before they could use the platform (21/66, 35.0%). The total SUS value was 66.55 (on a scale of 0-100), which is below the threshold of 68 for acceptability [26]. However, most of the users (45/59, 76.3%) positively recommended the platform with various degrees of enthusiasm.

### Element 3: Interviews With Users (GPs and Patients)

#### Participants

All participating GPs from the 3 practices that use the platform took part in the interviews (n=4; average age 46 years; 3 females; average interview duration 53 minutes) together with their patients who use the platform (n=15; average age 63 years; 9 females; average interview duration 32 minutes). The chronic conditions of users were similar to those of nonusers, with most suffering from cardiovascular and pulmonary chronic conditions. Some had more than one chronic disease. Interview results are described by topic and summarized in Table 2.

**Table 2:** Overview of topics and themes concerning the platform, as identified by questionnaire and interview.

Interview topics and themes		Examples
**Overall opinions**		
	Factors that may influence use	Viewpoint of GP^a^
	Recommendation to other patients	Not hypochondriac and must be digitally literate
	Recommendations for the platform	Personal health environment and need to expand before it is efficient
**User experience and needs**		
	Use of other features	Interest in other features (eg, scale, Fitbit, and glucose meter)
	Learning process for using the platform	Learning how to use the platform
	User friendliness	Easy interface
	Satisfaction measuring tool	Three easy-to-use measurements
	Continued use of the platform	Intent to continue using the platform
**Advantages**		
	Time and task efficient	Workload reductions
	Increased quality	More insight and involvement
	Increased accessibility	Lower threshold for consultation
	Self-management	More autonomy and empowerment
**Limitations**		
	Fixed time	Felt rigid and strict
	Technical questions	Difficulty using Bluetooth
	Lack of communication	Sometimes a bit impersonal
	Lack of feedback	Difficult to be motivated without a reaction

^a^GP: general practitioner.

#### Time and Workload for Patients and GPs

A GP indicated that patient-centered care led to more efficient care and could be supported by digital health. This can improve quality and accessibility, moving patients from a passive role to active self-management. All participating GPs indicated that the traditional model was no longer feasible due to high workloads, with one stating that the platform increased efficiency.

I think it is also quite easy for the doctor because all is arranged. ...they automatically receive a message about how the week went, and [if needed] they can call the patients or intervene.

GP

One reason for the decreased workload was that improved self-management by patients can save time for GPs.

...it takes less time, so you can do more. Yes, you can do it with fewer people. How many exactly? Yes, that needs to be determined by experience. Time will tell that, but this does relieve the workload; that is obvious.

GP

Of course, it saves a lot of time. You know the great thing about this way of working? You let the patient do a lot themselves and you lose the ‘noise’ from having to perform routine checks...which if it is done well...actually takes 5 minutes.

GP

Although digital health saves on travel time and expenses for GPs and patients, its implementation requires an initial time investment to become familiar with the system. However, patients did state that GPs responded faster and could be contacted more easily.

I much prefer that I can reach my doctor with an e-consultation, or [that] when I call, I can schedule a call-back time. This is better than when I am on hold for half an hour.

Patient

When I have finished a weekly measurement, it goes right to the GP...[and they]...see it...immediately. So, it is a lot faster. You need fewer steps to get to the goal. So, I think that’s a very big advantage.

Patient

Despite these positive experiences, some GPs warned that the system can increase workload. The contact options for patients can generate reminders and administrative actions. For example, one explained that she was unable to answer all the messages generated by patients. Some patients also used the platform more than needed, which GPs thought could increase workloads.

People check very often, so we may have to do something with that...because of course...you don’t have to measure your blood pressure every day.

GP

#### Personalized Care

Where time was saved by GPs using the platform, it could be used to improve patient-centered health care by allowing GPs to afford patients additional support.

Performing a hypertension check is of course not interesting...for the patient [or] the doctor. So that does not contribute much to your...relationship. But if you have a difficult period in your life, for whatever reason, and you just had several conversations and contacts with your doctor during that period, then you build a bond.

Patient

Although most patients and GPs experienced the advantages of personalized care through the platform, there were also a few who experienced the tool and digital health in general as more impersonal than traditional care.

The human dimension is becoming less and less important in health care. People must justify everything. A person is a person...not a machine. So yes, the interaction is also lessening...deteriorating.

Patient

#### Quality of Care

Opinions regarding care quality varied, with both positive and negative effects mentioned. Repeated monitoring by patients can help them feel seen by their GP, creating a sense of safety when GPs intervene because values indicate poor disease control. Patients with chronic illness who require support but never visit the practice can also be reached more easily because the platform is more accessible. Measurements are also more accurate because they are taken frequently, over a longer period, and at home, minimizing the potential stress and inconvenience of testing in a clinical setting. Prevention is another advantage of digital health. GP visits can become more efficient because evaluation has already started.

My doctor can monitor me without me noticing. You can have a precautionary look with each other, based on numbers, before having an appointment. I think that’s a big advantage.

Patient

#### Accessibility of Health Care

Patients and GPs were generally positive about the digital platform, viewing it as the future of health care. Having the support of GPs was crucial to implementing change because all patients initially started to use the platform based on a GP’s recommendation. When patients were actively involved with their GP, they tended to be enthusiastic about the platform. Patients felt the platform improved accessibility.

Because it’s just a lot easier. It’s fast, simple, and works well.

Patient

I can start using those questionnaires...I find it useful that it can be done remotely, even in a non-Corona era; I think it is just practical.

Patient

The digital skills of patients also affected accessibility, but patients typically had no problems.

Once you have it installed on your phone...it is not that difficult.

Patient

Physicians added that frail patients may not be able to take full control, but that monitoring should be possible in all cases. Technical problems could also affect accessibility to digital health services. For example, patients had difficulty using Bluetooth and synchronizing with the blood pressure monitor. They indicated that it sometimes worked and sometimes did not.

When I switch on my iPad, it synchronizes to the blood pressure monitor. Then it indicates that synchronization was successful and includes the result in a graph. [The last few times]...something has gone wrong in that process.

Patient

Most agreed that the platform was very user-friendly, with an interface that was clear and easy to use.

What I have used was quite simple, I must say.

Patient

I think it is very good. I can quickly look up patient data.

GP

However, patients wanted better instruction and communication about services, with some not knowing why a device was being used or who to approach with queries.

#### Self-management by Patients With Chronic Illness

It was noted that the platform requires that patients take an active role in managing their disease, which can represent an important change.

The patient starts to work on his own health, hopefully. That’s the idea. I really hope that it will give patients more insight into their disease and perhaps improve control.

GP

It is demanding for the patient [who] must understand what it is about and that it is best if he is also in charge of it himself. It is no longer what you [the GP] are going to do about it, but what [he] can do about it [him]self?

GP

GPs believed that patients could take this role but that some do not want to and will not benefit from the platform. Therefore, patients must show enthusiasm to be deemed eligible. Care must also be taken to exclude patients considered vulnerable, known to be illiterate, or with intellectual/cognitive limitations who lack the skills needed for self-management, specifically through a digital platform.

Patients generally agreed on the importance of self-management and empowerment.

You do get a picture of how you are doing. Especially if your blood contains too much sugar...it is very good to be able to check that yourself. Yes, I think that is important...it is very good if you can control that yourself. I always say you are responsible for your own health.

Patient

## Discussion

### Principal Findings

There was general agreement that the digital platform offered benefit from reducing time commitments and workloads for patients and GPs when used with appropriately selected patients. The quality and accessibility of care improved through greater patient-centeredness and a lower threshold for contact through different channels, respectively. Importantly, the self-management required by the tool increased patient autonomy and empowerment, and users considered the platform to be feasible, being both usable and easy to learn to use. However, they reported some minor technical issues (eg, Bluetooth connectivity) and wondered if people with fewer digital skills could manage.

It was notable that patients who had not used the platform but who had received the explanation were least positive about this new approach to chronic care management. Although they were relatively experienced and skilled with digital media, only a minority of the nonusers would recommend the platform due to concerns over data security and handling. This might be explained by there being less trust in digital health interventions when they have not experienced the tool themselves [27]. Among platform users, the percentage who would recommend the platform increased to approximately 76%, although the SUS score of 66.55 was just below the score of 68 required to claim success [28]. Active patient involvement in new health initiatives can decrease hesitation toward innovations and contribute to higher acceptance and support of a digital tool that may offer new insights and introduce new elements [29].

From the results of the IPQ-K, it can be interpreted that the study participants typically had a good understanding of their complaints with the impression that treatment could affect their health although it would probably stay with them for the rest of their lives. Compared to other populations with chronic diseases, they score relatively high but this is probably related to the fact the study population has relatively limited levels of multimorbidity as they are still supervised in the primary care setting [30]. However, the results from the SF-12 show that around half of the population is prevented from accomplishing the work and activities they would like to due to their physical and emotional health. This also reflects that the majority expresses impact on their energy levels and mood, which is often a risk in populations with chronic diseases [31,32]. Most respondents reported being digitally literate, which is not surprising because this first version of the platform was considered unsuitable for highly vulnerable patients. As with most digital innovations, we first introduced it among people who are both digitally and health literate [33]. Although users did not know all the available functions, they did tend to find the platform easy to use. Further analysis of what these users require, possibly with better education about what is already included, could improve utility among IT literate populations with less severe chronic illness. This endeavor will hopefully lead to greater awareness of the needs of less digitally or health literate patients with chronic illness who are so often forgotten [34].

An important element to take into account is that the platform started in April 2020 when Covid stressed the relevance of distance monitoring of patients and worked as a catalyzer for health care organizations to implement digital health, and more specifically telemonitoring, among vulnerable and chronically ill populations [35]. This definitely has positively influenced the uptake of the platform.

Opinions were divided regarding platform expansion. While GPs indicated that communication with other parties and improved data linkage to the GP information system was possible, patients were either neutral or had mixed opinions. Some patients wanted a personal patient environment, but others thought that the system was complicated enough. One GP agreed with this, elaborating to state that the platform should be simplified to have fewer functions and include less information, in line with the need for simplicity in use and scalability for digital health interventions in different settings [36]. This might also be further elaborated on when using the tool in populations with a lower digital literacy.

Patients saw that the digital platform brought many benefits, including improved efficiency, time savings, better care, and greater personal control over their illness. By contrast, GP opinions were more divided about whether the platform leads to preoccupation with illness and even restlessness, with some expressing concern that self-management changes the doctor-patient dynamic. This has been seen in similar interventions in neighboring countries [37]. It would be worthwhile to explore further what specific groups of patients with chronic illness are least and most suited for this type of platform.

Potential obstacles included the fixed time at which measurements were required, lack of communication and feedback, impersonal nature, and technical problems. Feedback was considered particularly necessary to ensure that patients know they are not measuring for nothing. Indeed, feedback is essential to achieve behavior change or ensure that patients take their medications and measurements [38]. Moreover, users were positive about the platform during this development phase, but they were neutral about whether they will use it regularly. This will depend not only on whether the platform is part of their treatment but also on the support and enthusiasm of the GP and their practice. While there is a responsibility for patients to use the platform, there is an equal responsibility for practitioners to keep the platform relevant. The role of the GP in these innovations should not be underestimated [39]. They also play an important role in influencing patients on the levels of perceived benefits and expected improvements in outcomes in the health belief model and UTUAT, which is also shown in other studies to be beneficial for adopting digital innovations [40].

GPs considered digital health to be the future, offering a good solution for organizing care more efficiently and positively influencing the doctor-patient relationship, as suggested elsewhere [41]. However, some patients considered the platform impersonal and were neutral about its impact on their relationship with the doctor. GPs thought that patients who avoid in-person care may have a lower threshold for using the platform than seeking in-person consultations and that any measurements will be more accurate. Although monitoring was considered possible for every disease group, it was mentioned that patients must be enthusiastic and have the necessary digital skills.

GPs were also concerned about the initial time investment and possible increased workload. This is an important consideration given that they need to be motivated. Similar studies have found that enthusiastic GPs can realize the extra impact on their work [42], but as in this study, they anticipate that benefits will come later. An important consideration in this is the tipping point at which an innovation used by a large group starts to become more efficient [43]. Current nonusers indicated that they would like to be notified when results are good, which could be a problem when a larger number of patients use the platform and are monitored. Handing control to patients could lead to the valid concern of actionable items increasing for GPs [42], with uncertainties about where to draw the line for blood pressure results and how to identify patients who are not completing the questionnaires correctly (or who do so too often). Another important element for motivation and reduction of perceived workload is that the integration of these kind of platforms with existing electronic health records should be very smooth [44].

### Recommendations to Remove Barriers and Facilitate Implementation

Some barriers and potential facilitators related to implementation are detailed in Table 3. Patients need to understand how a given digital platform works. Information videos or clear and simple instructions could offer value, especially for patients who cannot see the potential benefits of the platform. Patients require a clear explanation of when they must measure, when they must stop, why it is necessary, when they have to contact the GP again, and whether the GP has access to their data. GPs and patients may be more likely to use the digital platform if they can add or remove features as needed. For example, a GP may wish to remove some features, while a patient may want to use different measuring instruments (eg, a Fitbit). Here, flexibility may be key.

**Table 3:** Recommendations to facilitate implementation and remove barriers.

Recommendation	Example
Ensure that there is sufficient information about the digital platform, its use, and the goals.	A manual or introductory video can help.
Be clear about what information the health care provider can view and when contact will occur.	Create standardized messages and discuss uncertainties before starting.
Avoid fixed measurement times.	This could be resolved by giving patients control over when to take measurements.
Make the platform adaptable to the users.	Certain functions could be added or removed from the user’s personalized online environment.
Ensure balance between digital and in-person consultations to ensure that the platform is not experienced as impersonal.	Try to provide consultations at regular levels, either in person, by phone, or via the app, depending on the patient.
Start using the platform with patients who are less digitally and health literate to ensure that the whole population can benefit from the tool.	The population currently using the tool is clearly interested and equipped to work with such tools, but we must ensure applicability to all groups with additional research.

GPs indicated that it was useful to meet with a patient first to explain the process, but they also expressed concern that this can be time consuming. Both GPs and patients wanted medical questions to be resolved by the GP and technical questions to be resolved by the platform developer, requiring clarification on how the developer can be reached. Clear communication between the patient and GP also appears necessary, possibly with quarterly or yearly check-ups or a method to communicate when measurements are above or below that expected. Similarly, offering a standardized message when readings are within reference limits could prove beneficial, as has also been shown in the literature [44]. Functions must be adaptable to the patient, making it possible to take measurements on their own schedule to remove this obstacle and increase autonomy. Of course, measurement timings will still need to be specified (eg, 12 hours apart, every morning) based on clinical need and relevance, like measuring 1 week quarterly instead of daily the whole year round [45]. Given that patients in this study thought that digital health could appear impersonal, a balance will need to be struck with in-person consultations.

### Strengths and Limitations

The major strength of this study is that various groups gave feedback on our digital platform for chronic care, with patient users and nonusers and GPs being involved in the implementation. This gives an interesting insight into how the attractiveness and interest for the digital health platform differs among the 3 groups. Another strength is that we used triangulation by evaluating experiences on the platform through questionnaires and interviews. However, there were some important limitations. First, there was the potential for bias in the questionnaires received from patients within the health care practice. It could be that both the patients and the participating GPs had greater motivation than their peers in the general population, an issue that could affect the generalizability of our findings. Second, the interviews took place early in the implementation, so they may need to be repeated to see if there have been any developments. This will also be needed because the intervention will be adjusted based on this feedback to improve not only the platform and its implementation but also the outcomes and experiences of all participants.

### Conclusion

This study highlights the benefits of a digital platform for chronic illness in primary care along with the barriers and facilitators experienced by users. Both GPs and patients see this as an innovative tool that represents a good development improving care by offering time savings, greater efficiency, and greater patient-centeredness through improved autonomy and empowerment. Users considered the fixed measurement schedules, impersonal nature, time investment in learning and use, and poor communication and feedback to be the main barriers. Based on these findings, several recommendations have been developed that might improve the implementation of this or similar platforms in the future. Research must now focus on how to ensure that all patients gain benefit.

## Appendix

Multimedia Appendix 1 Original questionnaire in Dutch.

## Abbreviations

GP: general practitioner
IPQ-K: Illness Perception Questionnaire–Short
IT: information technology
SF-12: Short Form Survey–12 Item
SUS: System Usability Scale
UTUAT: unified theory of acceptance and use of technology
