# Supplementary material for: Evaluation of a Digital Self-management Platform for Patients With Chronic Illness in Primary Care: Qualitative Study of Stakeholders’ Perspectives
Source: JMIR Form Res. 2022 Aug 3;6(8):e38424. doi: 10.2196/38424 (PMC9386583; doi:10.2196/38424)
Supplement: Multimedia Appendix 1 [file formative_v6i8e38424_app1.pdf]

Klanttevredenheidsonderzoek Emma platform

Survey Flow

Block: Introductie (24 Questions)

Page Break

Start of Block: Introductie

Q14

Welkom bij dit onderzoek,

Dit onderzoek wordt uitgevoerd door de Rijksuniversiteit Groningen in opdracht van de huisartsen die gebruik maken van het Emma platform. Het doel van dit onderzoek is om het platform aan te passen op basis van uw mening en ervaring.

Het onderzoek zal starten met het invullen van een toestemmingsformulier. Wij zijn wettelijk verplicht om deelnemers aan wetenschappelijk onderzoek zo'n formulier te laten invullen.

Uw antwoorden zijn waardevol voor uw huisarts, voor de makers van het Emmaplatform en de toekomstige gebruikers van het platform.

Page Break

Q20
Voor dit onderzoek zijn we verplicht u een toestemmingsformulier te laten invullen. Wilt u de onderstaande uitspraken doorlezen?
Ik begrijp dat mijn naam alleen wordt gebruikt voor de toestemmingsverklaring. Ik begrijp dat de uitkomsten van de vragenlijst anoniem worden verwerkt en deze mogen worden gebruikt tot 15 jaar na het onderzoek volgens de huidige wetgeving. Ik begrijp dat de vragenlijstgegevens zo worden opgeslagen dat ze niet herleidbaar te zijn tot mij. Ik begrijp dat ik mijn medewerking aan dit onderzoek kan stoppen op ieder moment en zonder opgave van reden. Ik verklaar dat ik geheel vrijwillig mee doe an dit onderzoek. Ik geef toestemming om mijn gegevens te gebruiken voor de doeleinden van dit onderzoek Ik verklaar dat ik door middel van de gegeven informatie op duidelijke wijze ben ingelicht over de aard, methode en doel van het onderzoek.

○ Ja, ik ga akkoord met deze uitspraken (1)

○ Nee, ik ga niet akkoord met deze uitspraken (2)

Display This Question:

If Voor dit onderzoek zijn we verplicht u een toestemmingsformulier te laten invullen. Wilt u de ond... = Nee, ik ga niet akkoord met deze uitspraken

Q21 Als u bij de vorige vraag nee hebt ingevuld kunt u helaas niet meedoen aan dit onderzoek.

○ Nee ik wil niet mee doen aan het onderzoek. (1)

○ Ja ik wil toch wel mee doen aan het onderzoek. (2)

○ Ik wil graag meer informatie van de onderzoekers. (3)

Skip To: End of Survey If Als u bij de vorige vraag nee hebt ingevuld kunt u helaas niet meedoen aan dit onderzoek. = Nee ik wil niet mee doen aan het onderzoek.

Display This Question:

If Als u bij de vorige vraag nee hebt ingevuld kunt u helaas niet meedoen aan dit onderzoek. = Ik wil graag meer informatie van de onderzoekers.

Q22 Click to write the question text

○ Als u hier uw emailadres invult dan neemt de onderzoeker contact met u op. (5)
____

Page Break

Q15 Wilt u de volgende gegevens invullen voor de toestemmingsverklaring?

- ○ Naam (4) ____
- ○ Woonplaats (5) ____
- ○ Datum van vandaag (dd/mm/jjjj) (6) ____

Page Break

Q1
De Ziekteperceptie vragenlijst (IPQ-K).

Gaat u bij de volgende 9 vragen uit van de aandoening/ziekte waarvoor u het Emmaplatform gebruikt.

Vraag 1: 0 betekent "helemaal geen invloed", 10 betekent "zeer veel invloed

	0 (1)	1 (2)	2 (3)	3 (4)	4 (5)	5 (6)	6 (7)	7 (8)	8 (9)	9 (10)	10 (12)
Hoeveel beïnvloeden uw klachten uw leven? (1)	◯	◯	◯	◯	◯	◯	◯	◯	◯	◯	◯

Q25 Vraag 2: 0 betekent "een zeer korte tijd", 10 betekent "heel mijn leven"

	0 (1)	1 (2)	2 (3)	3 (4)	4 (5)	5 (6)	6 (7)	7 (8)	8 (9)	9 (10)	10 (12)
Hoelang denkt u dat uw klachten zullen duren? (1)	◯	◯	◯	◯	◯	◯	◯	◯	◯	◯	◯

Q26

Vraag 3: 0 betekent "helemaal geen controle", 10 betekent "zeer veel controle"

	0 (1)	1 (2)	2 (3)	3 (4)	4 (5)	5 (6)	6 (7)	7 (8)	8 (9)	9 (10)	10 (12)
Hoeveel controle heeft u over uw klachten? (1)	○	○	○	○	○	○	○	○	○	○	○

Q27

Vraag 4: 0 betekent "helemaal niet", 10 betekent "zeer veel"

	0 (1)	1 (2)	2 (3)	3 (4)	4 (5)	5 (6)	6 (7)	7 (8)	8 (9)	9 (10)	10 (12)
Hoe denkt u dat uw behandeling kan helpen bij uw klachten? (1)	○	○	○	○	○	○	○	○	○	○	○

Q28

Vraag 5: 0 betekent "helemaal geen beperking", 10 betekent "veel beperkingen"

	0 (1)	1 (2)	2 (3)	3 (4)	4 (5)	5 (6)	6 (7)	7 (8)	8 (9)	9 (10)	10 (12)
Hoe sterk ervaart u berperkingen door uw klachten? (1)	○	○	○	○	○	○	○	○	○	○	○

Q29

Vraag 6: 0 betekent "helemaal niet bezorgd", 10 betekent "zeer bezorgd"

	0 (1)	1 (2)	2 (3)	3 (4)	4 (5)	5 (6)	6 (7)	7 (8)	8 (9)	9 (10)	10 (12)
Hoe bezorgd bent u over uw klachten? (1)	○	○	○	○	○	○	○	○	○	○	○

Q30

Vraag 7: 0 betekent "helemaal geen begrip", 10 betekent "zeer veel begrip"

	0 (1)	1 (2)	2 (3)	3 (4)	4 (5)	5 (6)	6 (7)	7 (8)	8 (9)	9 (10)	10 (12)
In welke mate vindt u dat u uw klachten begrijpt? (1)	○	○	○	○	○	○	○	○	○	○	○

Q31

Vraag 8: 0 betekent "helemaal geen invloed", 10 betekent "zeer veel invloed"

	0 (1)	1 (2)	2 (3)	3 (4)	4 (5)	5 (6)	6 (7)	7 (8)	8 (9)	9 (10)	10 (12)
Hoeveel invloed hebben de klachten invloed op uw stemming? (b.v. maken de klachten u boos, bang, van streek of somber?) (1)	○	○	○	○	○	○	○	○	○	○	○

Page Break

Q2 Vraag 9

Noem alstublieft de drie belangrijkste factoren die naar uw mening uw klachten hebben veroorzaakt. In volgorde van belangrijkheid (dus op nummer 1 de belangrijkste, enzovoort).

De drie belangrijkste oorzaken zijn voor mij:

- ○ 1 (1) ____
- ○ 2 (2) ____
- ○ 3 (3) ____

Page Break

Q3
Deel 2 gezondheidsgerelateeerde kwaliteit van leven (SF-12).

De volgende vragen richten zich vooral op de invloed van uw gezondheid op uw dagelijks leven. Probeer bij elke vraag het antwoordt te selecteren wat het dichtste bij uw gevoel in de buurt komt.

De eerste vraag gaat over uw gezondheid in het algemeen.

	Slecht (1)	Redelijk (2)	Goed (3)	Zeer goed (4)	Uitstekend (5)
Hoe zou u over het algemeen uw gezondheid noemen? (1)	○	○	○	○	○

Q4 De volgende 2 stellingen gaan over activiteiten die u op een gewone dag zou willen doen.

Wordt u beperkt in het uitvoeren van deze activiteiten vanwege uw gezondheid?

	Ja, ernstig beperkt (1)	Ja, een beetje beperkt (2)	nee, helemaal niet beperkt (3)
Matige inspanning (zoals het verplaatsen van een tafel, stofzuigen, zwemmen of fietsen). (1)	○	○	○
Een paar trappen lopen? (3)	○	○	○

Page Break

Q5 De volgende 2 stellingen gaan over de invloed van uw gezondheid op uw werk en of andere dagelijkse activiteiten.

Hoe vaak heeft u in de afgelopen 4 weken 1van de volgende problemen bij het uitvoeren uw werk of andere dagelijkse bezigheden gehad, ten gevolge van u lichamelijke gezondheid?

	Atijd (2)	Meestal (3)	Soms (6)	Zelden (7)	Nooit (8)
U heeft minder bereikt dan u zou willen. (1)	○	○	○	○	○
U was beperkt in uw werk of andere bezigheden. (2)	○	○	○	○	○

Q6
De volgende 2 stellingen gaan over de invloed van uw mentale gezondheid op uw werk en/of andere dagelijkse activiteiten.

Hoe vaak heeft u in de afgelopen 4 weken een van de volgende problemen ondervonden bij uw werk of andere dagelijkse bezigheden ten gevolge van emotionele problemen (zoals depressieve of angstige gevoelens)?

	Altijd (1)	Meestal (2)	Soms (3)	Zelden (4)	Nooit (5)
U heeft minder bereikt dan u zou willen. (1)	○	○	○	○	○
U deed uw werk of andere bezigheden niet zo zorgvuldig als gewoonlijk. (2)	○	○	○	○	○

Page Break

Q7 De laatste stelling van dit gedeelte gaat over de mate waarin pijn een rol speelt in uw leven. Met pijn wordt bedoelt pijn als gevolg van uw klachten waarvoor u het platform gebruikt.

	Helemaal niet (1)	Klein beetje (2)	Nogal (3)	Veel (4)	Heel erg veel (5)
In welke mate bent u de afgelopen 4 week door pijn gehinderd in uw normale werk? (zowel werk buitenshuis als huishoudelijk werk) (1)	○	○	○	○	○

Q8
De volgende 4 vragen gaan over hoe u zich voelt en hoe het met u ging de afgelopen 4 weken. Wilt u bij elke vraag het antwoord selecteren dat het best past bij hoe u zich voelde.

Hoe vaak gedurende de afgelopen 4 weken....

	Altijd (1)	Meestal (2)	Soms (3)	Zelden (4)	Nooit (5)
Voelde u zich rustig en tevreden? (1)	○	○	○	○	○
Voelde u zich somber en neerslachtig? (2)	○	○	○	○	○
Had u veel energie? (3)	○	○	○	○	○
Had uw lichamelijke gezondheid of emotionele problemen u gehinderd bij uw sociale activiteiten (zoals vrienden of familiebezoeken, etc.)? (4)	○	○	○	○	○

Page Break

Q9

System usability scale:

De volgende 10 stellingen gaan over het gebruik van het Emma platform. Bij elke stelling geeft u aan in welke mate u het met de stelling eens of oneens bent.

	Helemaal niet mee eens (1)	Gedeeltelijk mee oneens (2)	Neutraal (3)	Gedeeltelijk mee eens (4)	Helemaal mee eens (5)
Ik denk dat ik het Emma platform regelmatig ga gebruiken. (1)	○	○	○	○	○
Het Emma platform is onnodig ingewikkeld. (2)	○	○	○	○	○
Het platform is gemakkelijk te gebruiken. (3)	○	○	○	○	○
Ik denk dat ik technische ondersteuning nodig heb van een ervaren gebruiker van het Emma platform om in staat te zijn het Emma platform te gebruiken. (4)	○	○	○	○	○
De verschillende functies van het Emma platform zijn goed geïntegreerd. (5)	○	○	○	○	○
Ik vond dat er veel tegenstrijdigheden in het platform zaten (6)	○	○	○	○	○
Het leren omgaan met het Emma platform zal voor de meeste mensen snel gaan. (7)	○	○	○	○	○
Het gebruik van het Emma platform is te omslachtig. (8)	○	○	○	○	○

Tijdens het gebruik van het Emma platform voelde ik me zelfverzekerd. (9)	○	○	○	○	○
Om het Emma platform uiteindelijk in gebruik te nemen moest ik eerst veel dingen leren. (10)	○	○	○	○	○

Page Break

Q35

0 betekent "zeer onwaarschijnlijk", 10 betekent "zeer waarschijnlijk"

	1 (1)	2 (13)	3 (12)	4 (11)	5 (2)	6 (3)	7 (4)	8 (5)	9 (6)	10 (7)
Hoe waarschijnlijk is het dat u het platform zult aanbevelen aan andere patiënten? (1)	○	○	○	○	○	○	○	○	○	○

Page Break

Q12 U bent aan het einde gekomen van deze vragenlijst. Vriendelijk bedankt voor het invullen! Uw antwoorden worden gebruikt om het platform nog beter te maken.

We willen nog graag het platform met gebruikers bespreken om hun wensen en behoeften beter te begrijpen. Zou u hier aan mee willen werken? Het gesprek vindt telefonisch plaats op een voor u geschikt moment.

Zouden wij contact met u op mogen nemen om een afspraak te plannen voor een telefonisch gesprek?
Als bedankje ontvangt u voor deelname aan het gesprek een kleinigheidje.

- ○ Ja (1)
- ○ Nee (2)

Display This Question:

If U bent aan het einde gekomen van deze vragenlijst. Vriendelijk bedankt voor het invullen! Uw antw... = Ja

Q13
Fijn dat we u mogen bellen voor een gesprek. Zou u hieronder uw contactgegevens kunnen invullen?

Deze gegevens gebruiken we om u te benaderen voor een afspraak.

- ○ Naam (1) ____
- ○ Telefoonnummer (2) ____
- ○ Emailadres (3) ____

End of Block: Introductie
